# Fast-Response Non-Contact Flexible Humidity Sensor Based on Direct-Writing Printing for Respiration Monitoring

**DOI:** 10.3390/bios13080792

**Published:** 2023-08-07

**Authors:** Xiaojun Chen, Kanglin Ma, Jialin Ou, Deyun Mo, Haishan Lian, Xin Li, Zaifu Cui, Yihui Luo

**Affiliations:** 1 School of Mechanical and Electronic Engineering, Lingnan Normal University, Zhanjiang 524048, China; 2 Department of Mechanical & Electrical Engineering, Xiamen University, Xiamen 361102, China

**Keywords:** direct-writing printing, respiratory monitoring, flexible humidity sensor, GNPS/MWCNT composite material

## Abstract

Respiratory monitoring is crucial for evaluating health status and identifying potential respiratory diseases such as respiratory failure, bronchitis, and pneumonia. Humidity sensors play a significant role in this regard, and efforts are being made to improve their performance. However, achieving ideal sensor parameters such as sensitivity, detection range, and response speed is challenging. In this work, we propose a flexible preparation method for a double-layer humidity sensor using PDMS as a substrate and a GNP/MWCNT composite material as a sensor element. This sensor exhibits high sensitivity (1.4 RH-1), a wide detection range (20–90%), ultra-fast response (0.35 s) and recovery (2.5 s), high repetitiveness (500 cycles), good long-term stability, and excellent flexibility. Due to these advantages, this sensor has potential applications in real-time clinical and home medical care, such as accurate human respiratory monitoring and non-invasive skin humidity monitoring. Hence, this humidity sensor can be a powerful tool to monitor respiratory moisture levels for diagnosing and treating respiratory diseases effectively.

## 1. Introduction

The monitoring of human respiration is crucial for the early detection of diseases and the management of personal health [1]. However, traditional respiratory monitoring technologies can be uncomfortable or complicated, making them impractical for daily use [2,3,4]. Humidity is a clear indicator that can be used to monitor respiration, and high-performance humidity sensors can play a vital role in health assessments and disease predictions [5]. For example, patients with sleep apnea syndrome (SAS) often suffer from respiratory suspensions, while patients with pneumonia, bronchitis, and asthma frequently experience shortness of breath [6,7]. Monitoring respiratory frequency can be helpful in treating these conditions. By using humidity sensors for respiratory monitoring, health professionals and individuals can obtain vital information to maintain personal health and provide early interventions for respiratory illnesses [8].

In recent years, the development of flexible electronic technology has introduced new possibilities for respiratory monitoring [9]. As an important component of this technology, humidity sensors can monitor changes in the surrounding environment of the human body, which is beneficial for preventing and treating diseases. There are three types of conventional electrical-based humidity sensors, namely capacitance [10,11], resistance [12,13,14,15], and piezoelectric [16,17,18] sensors. Capacitive and piezoelectric sensors have insufficient stability compared with resistance sensors. They are highly susceptible to external interference, making them inappropriate for measuring high-humidity environments. The resistance-based humidity sensor offers superior advantages such as high sensitivity, a wide detection range, and fast response speed. It detects variations in resistance by using hygroscopic materials with micro/nano-structural properties, which has become a popular research topic. Cha et al. [19] present a flexible humidity sensor that utilizes a polyetheramine film characterized by high sensitivity and rapid response rates, making it suitable for human respiratory testing. Zhu et al. [20] propose a humidity sensor based on multi-wall carbon nanotubes and polypyrrole nanoparticles, which can monitor breathing under a wide range of humidity conditions. The sensor exhibits excellent sensitivity, response time, stability, and repeatability. Abed et al. [21] introduce a real-time human respiratory monitoring system that utilizes wireless humidity sensor arrays. The system can monitor respiratory frequency and depth by detecting moisture in exhaled gas, thus offering great potential for medical applications.

Humidity sensors available on the market have certain limitations with regards to their sensitivity, response, and detection range. For instance, Ghanem et al. [22] used a solitary-gel method to prepare a zinc oxide film on a glass substrate to develop a humidity sensor with a detection range of 15–95%RH and a resistance change of approximately 20 trillion euros, with a corresponding response time of 7 s at 100 °C. Although this sensor has a wide detection range, the hydrophobic nature of the zinc oxide film limits its sensitivity. On the other hand, titanium dioxide humidity sensors exhibit better hydrophilicity and sensitivity but have poor recovery performance, with a response and recovery time of 3 s and 50 s, respectively [23]. Hsueh et al. [24] propose a flexible humidity sensor using gallium oxide (GA2O3) and nanowires that have a rapid response time and wide measurement range, but their sensitivity is limited, and the sensor preparation process is complicated. Pi-Guey et al. [25] present a flexible humidity sensor using copper nanowires, which exhibits good stability and sensitivity but has a narrow measurement range that is applicable only in industrial settings. Thus, there is an urgent need to develop a non-contact flexible humidity sensor with a wide detection range, high sensitivity, and rapid response that can play a crucial role in monitoring human health.

Compared with traditional preparation methods [26,27,28], this work proposes the utilization of direct-writing printing techniques in the preparation of flexible humidity sensors that offer a wide detection range, high sensitivity, and repeatability, making them especially suitable for monitoring human health [29,30]. This humidity sensor employs a flexible PDMS substrate, with a GNP/MWCNT composite material serving as its sensor element. The composite material is composed of numerous micro-nano structures that form a three-dimensional conductive network. When exposed to moisture, the water molecules enable the formation of a conductive circuit through the three-dimensional conductive network, resulting in changes in resistance that precisely measure environmental humidity. Here, it demonstrates fast response, high sensitivity, good repeatability, and excellent long-term stability. This work presents a promising humidity sensor that can be used in multi-mode breathing monitoring, indicating its potential for application in the next generation of wearable/flexible devices for health monitoring.

## 2. Material and Method

### 2.1. Materials

The following were used in this study: Graphene nanoflakes (GNPs), dispersed solution (graphene nanoflake solution, MW = 12.01 g/mol, dissolved in H_2_O, concentration: 1 mg/mL); Multi-wall carbon nanotubes (MWCNT) (Sigma-Aldrich, Saint Louis, MI, USA, OD × L = 6–13 nm × 2.5–20 μm, carbon content: >98%); Polyethylene oxide (PEO) (average molecular weight: 3 million); Polydimethylsiloxane (PDMS) (Sylgard 184, Corning Inc., USA, composed of matrix and cross-linking curing agent); Electrode material HS-8200 Ag (Hanstars, Dongguan Haisi Electronics Co., Ltd., Dongguan, China).

### 2.2. Preparation of GNP/MWCNT Precursor Solution

MWCNTs and PEO were dispersed in the GNP dispersion solution in equal proportion to form the GNP/MWCNT precursor solution. The precursor solution was then placed on a magnetic stirrer and stirred at 150 r/min for 1 h. Afterward, it was subjected to ultrasonic treatment for 2 h in a water bath to uniformly mix the precursor solution and to avoid aggregation.

### 2.3. Preparation of PDMS Flexible Substrate

Mix the PDMS substrate and the crosslinking curing agent at a ratio of 12:1. After stirring at a speed of 200 r/min, treat it under vacuum at 4 °C for 2 h. Then, place it on the polished surface of a silicon wafer and spin-coat it using a spin-coater at a speed of 300 r/min for 3 min. Treat it under vacuum to remove air bubbles for 30 min in a vacuum oven and then keep it at 80 °C for 60 min. Cut the formed PDMS film (with a thickness of ~500 μm) into rectangular bases of length × width = 1.5 cm × 1 cm using a blade. Activate the surface of the rectangular base using plasma (Q150 Alpha Plasma, Alpha Plasma Asia, Rosenheim, Germany) for 3 min to increase the surface energy of the PDMS base and to enhance the adhesion between the electrode-sensitive unit and the surface of the PDMS substrate.

### 2.4. Direct-Writing Printing of Sensitive Sensor Units

A self-made 3D printing system is used to fabricate humidity-sensitive sensor units, which mainly consists of a direct-writing inkjet module, a high-precision Oxy motion platform (with a minimum movement resolution of up to 0.001 μm and a movement accuracy of ± 0.2 µm), a *Z*-axis motion platform, a heating-plate module, and an industrial CCD camera module. The direct-writing inkjet module is composed of a syringe pump (Harvard/11 Pico Plus, Harvard Instruments, Harvard, MA, USA) and a direct-writing nozzle. When the printing begins, the *Z*-axis motion platform is adjusted to bring the direct-writing nozzle to a distance of ~1 mm from the surface of the PDMS substrate. By setting a certain liquid discharge rate to the syringe pump, the direct-writing nozzle begins to emit liquid due to the extrusion pressure of the syringe pump. The high-precision motion platform is controlled by a computer to move according to the pattern. The heating-plate module on the Oxy motion platform is used for rapid solidification of the direct-writing pattern, and the heating temperature is set to 80 °C. The industrial CCD camera is used to observe the liquid emission and pattern formation of the direct-writing nozzle.

### 2.5. Characterization and Testing

Morphology characterization: The macroscopic appearance of the sensitive unit is observed using optical microscopy (MIT series, Chongqing Aote Optical instrument Co., Ltd., Chongqing, China); the contact angle of water droplets on the surface of the PDMS substrate is measured using a contact-angle measuring instrument (DSA100, Klux AG, Cologne, Germany); the microstructure of the sensitive unit is observed using scanning electron microscopy (JSM-IT500a, JEOL, Tokyo, Japan); the internal microstructure of the sensitive unit is examined using transmission electron microscopy (JEM-1400, JEOL, Japan); the structure of the wet-sensitive GNP/MWCNT unit is analyzed using an X-ray diffractometer (XRD) (Bruker D8-A25, AXS GmbH, Brucker, Germany).

Electrical- and humidity-sensing characteristics testing: A gas control system (GSL-3Z, Hefei Kejing Co., Ltd., Hefei, China) is used to control the entry of dry N2 or humid N2 into the chamber to regulate the humidity. The calibration of chamber humidity is performed using a precision humidity meter (Xima AR847, Taina Instruments, Shanghai, China). The flexible humidity sensor is placed in the chamber for testing, and data are collected and recorded using a multifunctional digital multimeter (Agilent 34410A, Agilent Technologies Co., Ltd., Santa Clara, CA, USA) and a computer.

## 3. Results and Discussion

### 3.1. Preparation of the Flexible Humidity Sensor

**Figure 1:**
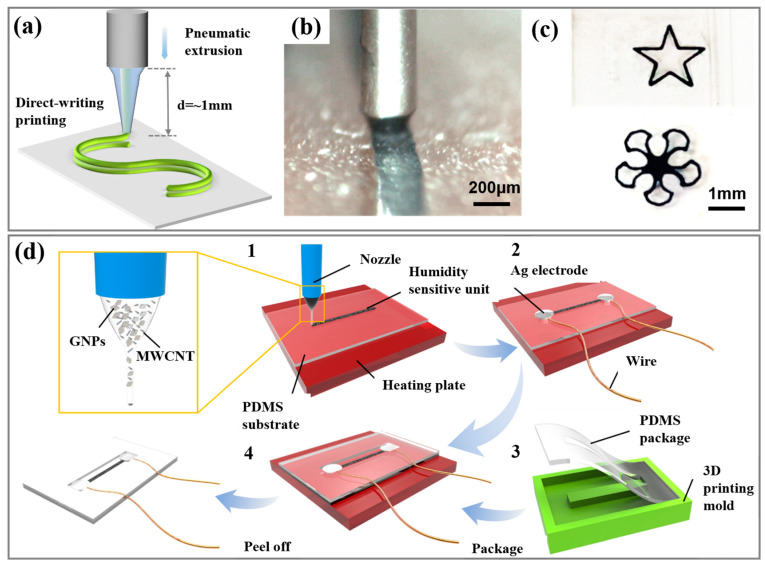
(**a**) Schematic diagram of the direct-writing inkjet printing principle. (**b**) Liquid ejection situation of the direct-writing inkjet printing head. (**c**) Optical images of different patterned structures. (**d**) Manufacturing process flow of the flexible humidity sensor.

The sensitive unit structure of the flexible humidity sensor is manufactured using a self-made direct-writing printing system (Figure 1a). When the direct-writing printing starts, the solution is quickly transported to the surface of the PDMS substrate, forming a micro-contact with the printing head and achieving patterned printing of the sensitive unit (Figure 1b). By controlling the repeated movement of the mobile platform, a multi-layer structure of the humidity-sensitive unit is printed. Various patterned direct-writing printings on the plane are fabricated, obtaining simple polygonal or complex curved patterns (Figure 1c). This indicates the advantage of quickly manufacturing different structural patterns based on actual needs. In the manufacturing of the flexible humidity sensor, conductive silver paste is coated onto both ends of the sensitive unit and bonded with leads as conductive electrodes, which is advantageous for subsequent electrical property tests. A 3D-printed PDMS template is used for reverse molding of the PDMS encapsulation layer. To expose the sensitive unit to air, a convex structure is designed in the middle of the PDMS template. Finally, the PDMS encapsulation layer and the PDMS substrate containing the sensitive unit are hot-pressed to complete the manufacturing of the double-layer flexible humidity sensor (Figure 1d).

**Figure 2:**
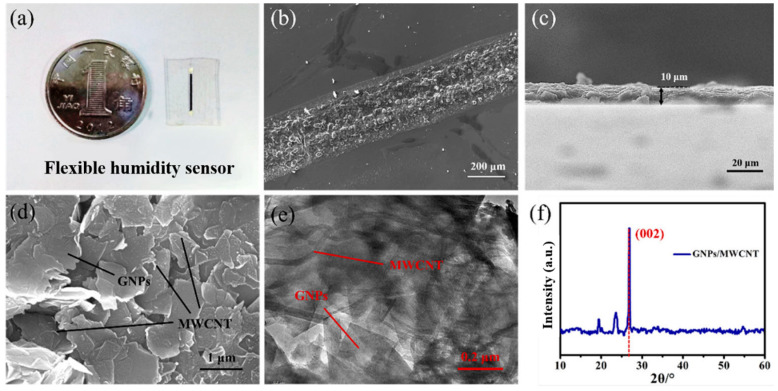
(**a**) Physical image of the linear-structured flexible humidity sensor. (**b**) SEM image of the humidity-sensitive unit from a top-down perspective. (**c**) SEM image of the humidity-sensitive unit from a side view. (**d**) SEM image of the GNP/MWCNT composite material. (**e**) TEM image of the GNP/MWCNT composite material. (**f**) XRD spectrum.

A flexible humidity sensor with a linear structure was manufactured by direct-writing printing (Figure 2a). The thickness of the entire device is ~1 mm, and the length of the sensitive unit is 8 mm. SEM images of the top view and side view of the humidity-sensitive unit after multiple depositions are shown in Figure 2b,c. The line width of the sensitive unit is 350 μm, and the height is 10 μm. The micro-surface of the sensitive unit is uneven and irregular in shape, which is conducive to increasing the contact between the sensitive unit and moisture and to improving the sensitivity of the sensor.

As shown in Figure 2d,e, MWCNTs are randomly dispersed among GNPs in the linear structure of the humidity-sensitive unit, forming bridging sections that provide an effective three-dimensional conductive network path for the humidity-sensitive unit. The printed GNP/MWCNT composite material maintains excellent dispersion without instability such as aggregation. The three-dimensional conductive network structure provides a transmission channel that enhances conductive stability. It provides a fast channel for electronic transmission to ensure the excellent conductivity of the humidity-sensitive unit. As shown in Figure 2f, the 2θ value of the GNP/MWCNT composite material displays a sharp peak centered at 26°, corresponding to the (002) plane of the GNPs. This indicates that GNPs were effectively prevented from aggregating in the polymer by MWCNTs. Due to the 2θ value being affected by the polymer at 19.5° and 23.5°, the XRD spectrum of GNP/MWCNT composite material has two small peaks at these positions [31]. Therefore, it is proved that MWCNTs successfully prevent the aggregation of GNPs and polymers, enabling the uniform dispersion of GNPs/MWCNTs and obtaining a humidity-sensitive unit with excellent conductivity and humidity sensitivity [32,33].

### 3.2. Surface Modification of the PDMS Substrate

**Figure 3:**
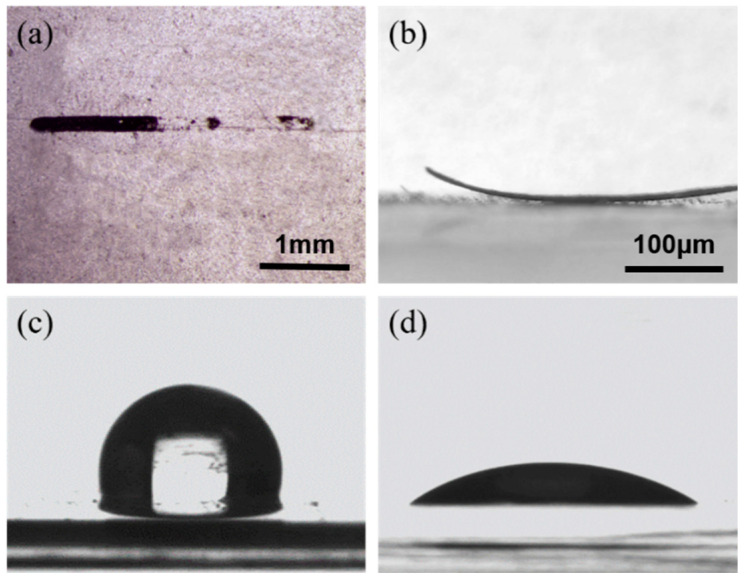
Humidity-sensitive unit: (**a**) Fall off; (**b**) Warm; (**c**) Contact angle without surface treatment; (**d**) Contact angle after surface treatment.

When using flexible devices in bending and stretching, the wet-sensitive material at the interface is prone to detachment from the substrate, leading to increased electrical resistance, drift, or even failure at the baseline of the device. Therefore, stable bonding between the sensitive unit and the flexible substrate is crucial to ensure the stability, accuracy, and reliability of the sensor performance. During the direct-writing printing process, the adhesion of the wet-sensitive unit on the PDMS substrate surface is poor. It is difficult to deposit the wet-sensitive unit stably on the surface of PDMS, and it is prone to detachment (Figure 3a). With the solidification and formation of PDMS, the wet-sensitive unit adhered to the surface of PDMS will detach or warp (Figure 3b). This is due to the influence of the -CH3 and -CH2 groups on the surface of PDMS, resulting in low surface energy and poor surface wettability after solidification. Therefore, surface modification of PDMS is necessary to enhance the bonding between the wet-sensitive unit and the PDMS substrate.

To enhance the adhesion of the PDMS substrate, plasma oxidation activation was employed to treat the PDMS surface. In Figure 3c,d, the contact angle of the treated PDMS surface was reduced from ~102.6° to ~26.5°, indicating a significant improvement in surface wettability. After plasma oxidation activation, the wet-sensitive unit could adhere tightly to the substrate and would not detach, even with slight abrasion. This is because plasma oxidation activation creates an activated surface on PDMS, which further reacts with active oxygen to partially remove the -CH3 and -CH2 groups from the surface and introduce many polar groups such as -OH and -COOH. These polar groups dramatically increase the surface activity of PDMS, allowing for a stable and strong connection between the PDMS surface and the wet-sensitive unit [34].

### 3.3. Performance of the Flexible Humidity Sensor

The linear flexible humidity sensor was placed in the self-built humidity detection system (Figure S1, supporting material). The change in the rate of sensor impedance ∆R/R with respect to relative humidity RH was investigated, and the sensitivity coefficient of relative humidity S_rh_ was calculated according to formula (1).

(1) $$S_{\mathrm{rh}}=\frac{\delta \left(\frac{\Delta R}{R}\right)}{\delta \mathrm{RH}}$$

∆R refers to the change in resistance value; R refers to the initial resistance value without pressure applied, and δRH refers to the resistance value with pressure applied.

**Figure 4:**
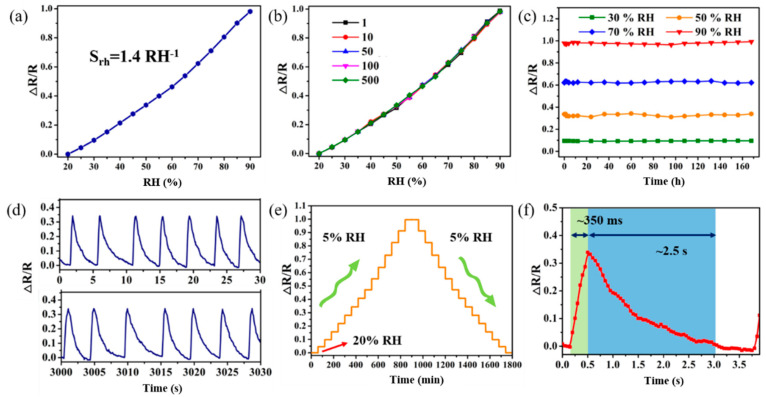
Performance characterization of the flexible humidity sensor. (**a**) Relative humidity sensitivity characteristics. (**b**) Repeatability test of relative humidity. (**c**) Humidity-sensitive stability test. (**d**) Humidity-sensitive cyclic testing. (**e**) ∆R/R changes with humidity gradient. (**f**) Step-response characteristics of humidity.

As shown in Figure 4a, the sensitivity coefficient of relative humidity Srh of the humidity sensor is as high as ~1.4 RH^−1^. As the relative humidity increases from 20% to 90%, ∆R/R increases from 0 to ~0.98, indicating that the change in relative humidity has a significant impact on the resistance of the sensor. That is, the resistance of the sensor gradually increases with the increase of humidity, which is consistent with the analysis of the humidity-sensitive mechanism.

Relative humidity was tested in increments of 10%RH, and 500 repeated tests were conducted as shown in Figure 4b. The results show that the coincidence of the sensor’s relative humidity sensitivity characteristic curve is extremely high, and the corresponding ∆R/R for each gradient relative humidity is almost similar. This proves that the humidity sensitivity of the sensor has good stability and consistency in the range of 20%RH to 90%RH. Continuous testing of the sensor was also conducted for 7 days at four relative humidity levels of 30%RH, 50%RH, 70%RH, and 90%RH, as shown in Figure 4c. The ∆R/R of the sensor at different relative humidity levels remained almost unchanged, indicating good stability.

By rapidly opening and closing the valve, drying N2 or N2 with moisture can be quickly introduced. Figure 4d shows the response characteristics of the sensor as the humidity changes from 60%RH to 80%RH. As the humidity environment in the chamber constantly changes, ∆R/R maintains good consistency between ~0 and ~0.35. At the same time, when the humidity drops to ~60%, ∆R/R also stabilizes to the initial state. Using 20%RH relative humidity as the starting point, the stability characteristics of the sensor to relative humidity were investigated within 1 h, with the relative humidity increasing or decreasing in increments of 5% (Figure 4e). The resistance of the sensor is consistent and stable at the same humidity, and its change trend with the humidity increase is obvious, with excellent consistency in response and recovery. The step-response characteristics of the sensor in the humidity change range from 60%RH to 80%RH were investigated, as shown in Figure 4f. When the humidity changes, the resistance value immediately responds. However, when the humidity decreases, the resistance value recovers relatively slowly. The sensitivity mechanism of humidity sensors is confirmed through these response characteristics. The humidity response/recovery time of the sensor is approximately 0.35 s and 2.5 s, respectively. Its humidity response time is a significant advantage (a comparison table summarizing the different humidity sensors is shown in Table S1, Supporting Materials), as it can quickly sense humidity changes and quickly feedback the humidity changes to the receiver. This indicates that the sensor has enormous potential for non-contact humidity monitoring.

**Figure 5:**
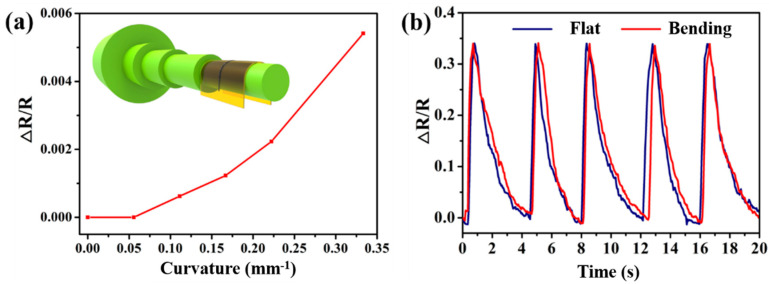
Characterization of bending deformation of the sensor. (**a**) Sensitivity characteristics under different curvatures. (**b**) Cyclic characteristics in the flat state and the mechanical bending state.

To evaluate the interference of bending deformation on the performance of the flexible humidity sensor, the response characteristics of the sensor’s bending deformation were tested at different curvatures. The linear flexible humidity sensor was attached to the surface of stepped shafts with different radii of 18 mm, 9 mm, 6 mm, 4.5 mm, and 3 mm (Figure 5a). As the curvature increased from 0 to 0.33 mm^−1^, the ∆R/R value increased from 0 to ~0.005. The bending deformation of the sensor almost does not have a significant impact on its resistance, indicating that the flexible humidity sensor is suitable for use in bent environments. Moreover, this characteristic makes the sensor’s accurate in surface adhering testing. As shown in Figure 5b, the response characteristics of the sensor were tested under conditions of relative humidity changes from 60%RH to 80%RH in the state of flat and mechanical bending with a bending radius of 6 mm. Before the test, the sensor sample was bent 500 times under mechanical bending conditions. The experimental results show that the humidity response of the sensor in the range from 60%RH to 80%RH is very similar in both the flat state and the bent state. The sensor shows periodic and repeatable response abilities, and the sensitivity of the sensor remains good after multiple bending.

### 3.4. Sensitivivity Mechanism of the Flexible Humidity Sensor

**Figure 6:**
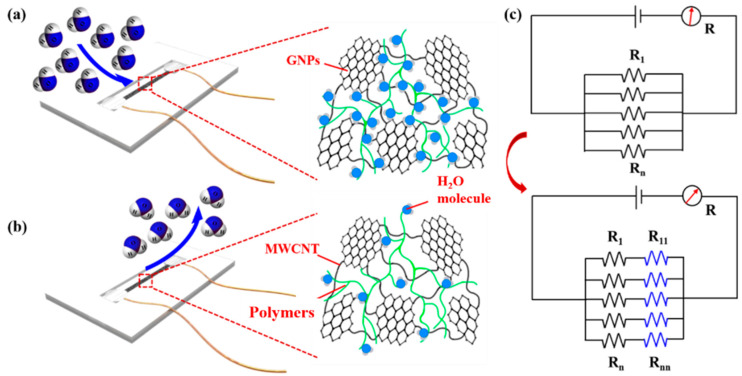
Humidity-sensitive mechanism model: (**a**) Normal humidity; (**b**) Increased humidity; (**c**) Circuit model of the humidity-sensitive unit.

The humidity-sensitive mechanism model is shown in Figure 6a,b. GNPs/MWCNTs are uniformly filled in the polymer to form a humidity-sensitive unit with numerous small gaps or polymer encapsulation and adhesion between them, exhibiting a strong adsorption capacity for water molecules. When the relative humidity increases, the gas flow brings in a large number of water molecules. These water molecules quickly aggregate in the small gaps between the GNPs/MWCNTs in the polymer. The filling of water molecules obstructs the conductive paths of the three-dimensional conductive network, resulting in a significant increase in resistance as humidity increases. When dry airflow removes the water molecules concentrated between the GNPs/MWCNTs, the resistance recovers [35]. An electrical circuit model is established as shown in Figure 6c. As humidity increases, the introduced water molecules connect new resistances (R11~Rnn) in series with the conductive paths, leading to an increase in resistance. Owing to the strong adsorption of the polymer for water molecules, the sensor exhibits a rapid response to changes in humidity. However, it also makes it difficult for water molecules to detach from between the GNPs/MWCNTs, resulting in poor recovery performance [36].

### 3.5. Application of Non-Contact Flexible Humidity Sensors in Respiratory Monitoring

It is well known that the human respiratory process involves periodically inhaling relatively dry air and exhaling relatively humid air, which can cause significant changes in the humidity around the nose or mouth. Flexible humidity sensors with a rapid response can play an important role, especially in respiratory monitoring. This type of sensor can measure the humidity in exhaled air to determine the breathing rate. The normal respiratory rate of adults ranges from 12 to 20 times/min, which is about 3 to 5 s per breath. Breathing rates that are too fast or too slow can have adverse effects on human health. A fast breathing rate can cause shallow breathing and hypoxia, affecting the heart rate and circulation, causing anxiety and tension, and worsening respiratory diseases such as asthma. A slow breathing rate can cause carbon dioxide retention and acidosis, affecting the nervous system and cognitive ability and causing issues such as dizziness and fainting. Therefore, using flexible humidity sensors for real-time monitoring of breathing rates is essential for maintaining good health.

**Figure 7:**
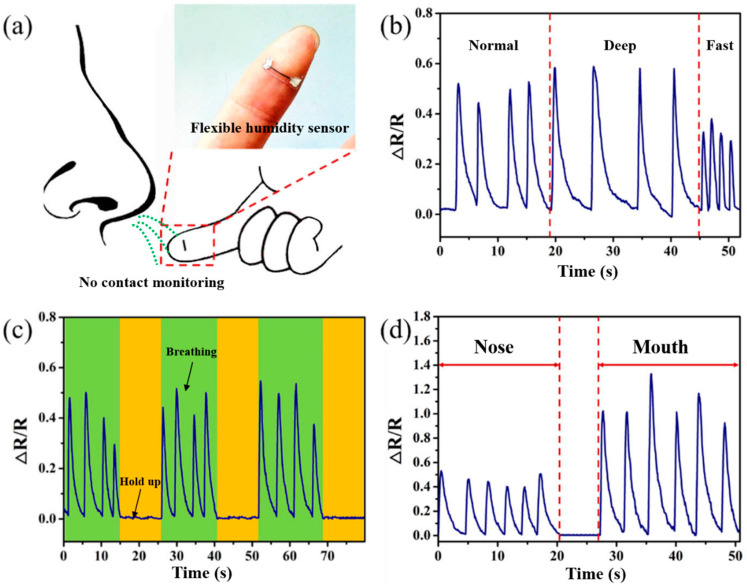
Demonstration of the applications of respiratory monitoring. (**a**) Schematic diagram of respiratory monitoring. (**b**) Monitoring of normal breathing, deep breathing, and rapid breathing states. (**c**) Monitoring of breathing and the breath-holding process. (**d**) Monitoring of nose breathing and mouth breathing.

As shown in Figure 7a, the flexible humidity sensor is attached to the finger first and then brought close to the nose to monitor the breathing in real time. The distance between the sensor and the nostrils is about 3 cm, and the gas from respiration can directly contact the sensor’s sensitive element. As shown in Figure 7b, the response curve of the sensor was tested under three different breathing states: normal breathing, deep breathing, and rapid breathing. It is evident that the three different breathing states can be quickly distinguished by the response frequency and ∆R/R value, where normal breathing is about 15 times/min, deep breathing is about 9 times/min, and rapid breathing is about 35 times/min. Here, the lower ∆R/R value during rapid breathing is due to the fast breathing rate, which causes the sensor to not be able to recover within one breathing cycle. However, the next cycle will have already begun, thereby increasing its initial resistance value. The periodic variation in resistance value under different breathing states demonstrates the repeatability of the sensor in respiratory monitoring applications.

The response of the sensor under normal breathing and breath-holding conditions was evaluated. As shown in Figure 7c, the sensor responds quickly during normal breathing, and when breath-holding, the ∆R/R value returns to the initial value and remains unchanged. This indicates that the sensor can clearly identify the signal of breath-holding, and the resistance of the sensor is almost constant in such a state. This demonstrates that the sensor has great potential for monitoring sleep apnea symptoms and respiratory arrest caused by diseases. In some special cases, such as during intense exercise and nasal congestion, people may also use their mouth to breathe. Therefore, we investigated the response characteristics of the humidity sensor for breathing through the mouth and nose, respectively (Figure 7d). The results showed that the relative humidity change caused by mouth breathing is greater than that caused by nose breathing. The sensor can monitor different breathing patterns, demonstrating its important role in monitoring mouth and nose breathing.

**Figure 8:**
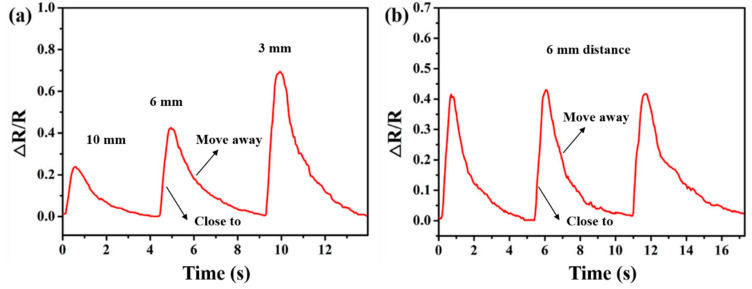
Demonstration of the non-contact monitoring of skin humidity. (**a**) Response at different distances. (**b**) Reproducibility test at the same distance.

As human skin can generate and distribute moisture, the sensor will respond promptly to changes in the surrounding humidity when the finger is close to the sensor. Figure 8a shows the responses of the sensor when the finger is at different distances. As the distance between the finger and the sensor changes from 10 mm to 6 mm and then to 3 mm, the distance between the finger and the sensor continuously decreases, and the resistance of the sensor significantly increases due to the increase in the surrounding humidity. When the finger is moved away, the resistance of the sensor will quickly recover until it returns to its original state. Figure 8b shows the repeatability test when the distance between the finger and the sensor is about 6 mm. It is obvious that when the finger remains at the same distance each time, the response of the sensor is consistent. This also proves that the sensor has good repeatability and reproducibility. This means that the sensor can achieve fast and accurate humidity detection without contact with the measured object. As the sensor has excellent humidity sensitivity, it has broad prospects for non-contact detection applications, such as drone control, humidity sensing, non-contact switches, and plant moisture measurement in the industrial field.

## 4. Conclusions

In this work, we have successfully demonstrated a flexible humidity sensor using direct-ink-printed GNPs/MWCNTs as the sensing electrode material and PDMS as the flexible substrate. The device features an ultra-fast response (0.35 s)/recovery (2.5 s), high sensitivity (1.4 RH-1), and high repeatability (500 cycles). Benefiting from these advantages, the device has been evaluated in monitoring human breathing patterns, such as rapid breathing, normal breathing, deep breathing, breathing and breath-holding, nose breathing, and mouth breathing. Additionally, we have verified the prospects of using this humidity sensor in non-contact detection. We believe that in the future, this sensor can be used to accurately sense abnormal human breathing behaviors, including suffocation, respiratory arrest, and asthma, thus playing an important role in human health monitoring.

## Data Availability

Not applicable.

## Supplementary Materials

The following supporting information [37,38,39,40,41,42] can be downloaded at: https://www.mdpi.com/article/10.3390/bios13080792/s1, Figure S1: Humidity detection system; Table S1: Summary of the characteristics of different humidity sensors.
