# Supplementary material for: Fast-Response Non-Contact Flexible Humidity Sensor Based on Direct-Writing Printing for Respiration Monitoring
Source: Biosensors (Basel). 2023 Aug 7;13(8):792. doi: 10.3390/bios13080792 (PMC10452166; doi:10.3390/bios13080792)
Supplement: Supplementary file 1 [file biosensors-13-00792-s001.zip › biosensors-2518243-supplementary.pdf]

Supporting material

Fast-Response Non-Contact Flexible Humidity Sensor Based on Direct-Writing Printing for Respiration Monitoring

Xiaojun Chen [1,*], Kanglin Ma [1], Jialin Ou [1], Deyun Mo [1], Haishan Lian [1], Xin Li [1], Zaifu Cui [1] and Yihui Luo [2]

1 School of Mechanical and Electronic Engineering, Lingnan Normal University, Zhanjiang 524048, China; mkl447314@163.com (K.M.); 18875943150@163.com (J.O.); dewin_mo@163.com (D.M.); lianhs@lingnan.edu.cn (H.L.); 19820180155649@stu.xmu.edu.cn (X.L.); zaifucui@lingnan.edu.cn (Z.C.)
2 Department of Mechanical & Electrical Engineering, Xiamen University, Xiamen 361102, China; 19920161151457@stu.xmu.edu.cn (Y.L.)
* Correspondence: chxj@lingnan.edu.cn (X.C.); Tel.: +86-180-3025-0016

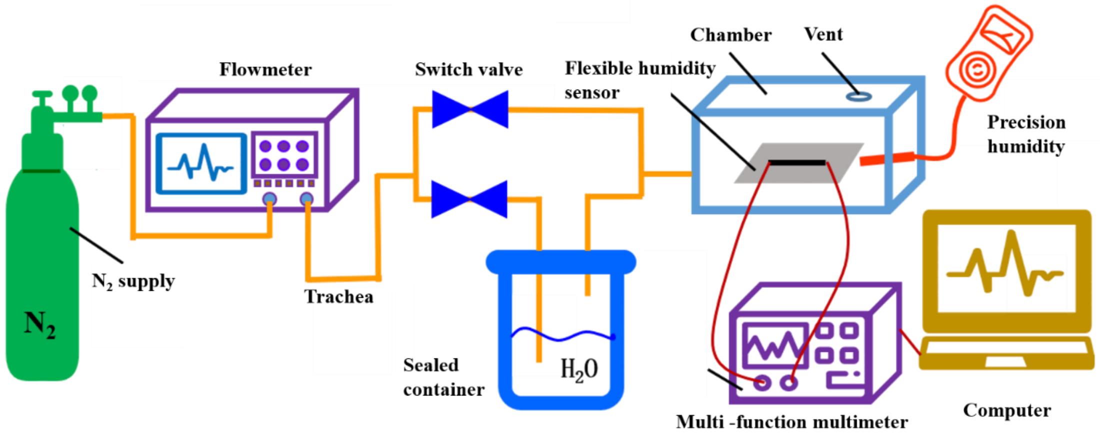

Figure S1. Humidity detection system.

Table S1. Summary of the characteristics of different humidity sensors.

Sensing type	Sensing material	substrate	Response/re-cover	sensitivity	Range of hu-midity
Resistive [1]	Silver nanowires	Polyimide	5 s/14 s	0.01 MΩ/%RH	11-95 %RH
Capacitive[2]	Graphene	Polyamide	1 s/1 s	57.36 pF/%RH	11-95 %RH
Resistive[3]	α-Fe2O3	Polyam-ide/polyure-thane	4 s/8 s	1.16 MΩ/%RH	10-98 %RH
Capacitive[4]	GO/PEDOT:PSS	Polystyrene	1 s/5 s	0.076 pF/%RH	5-95 %RH
Resistive[5]	Cellulose nano-fiber/carbon black	Ag	10 s/6 s	43.2 pF/%RH	30-90 %RH
Resistive[6]	Silk fibroin/silver	PET	73.1 s/11.3 s	0.2 nA/%RH	N/A
This work	GNPS/MWCNT	PDMS	0.35 s/2.5 s	1.4 RH-1	20-90%RH

Reference

[1] Yang, Y., Chen, Z., Lu, N., Xu, Q., Chen, G., Zhou, Y., Lu, J., Gu, C. & Fan, Z. (2019). A flexible humidity sensor based on silver nanowires and polyimide. Materials Research Express, 7(2), 025609.
[2] Khot, L.R., Nemade, K.R. & Ghotekar, S.K. (2020). Graphene-based room temperature humidity sensor for biomedical

applications. Journal of Materials Science: Materials in Electronics, 31(19), 16803-16810.
[3] Chakraborty, S., Pal, S. & Banerjee, D. (2021). Dandelion-like α-Fe2O3 microsphere coated thermoresponsive core-sheath electrospun nanofiber for high-performance humidity sensor. Sensors and Actuators B: Chemical, 334, 129688.
[4] Hossain, M.M., Kim, S., Kim, J.H. & Lee, J.J. (2020). Self-Powered Triboelectric Nanogenerator-Based Humidity Sensor Fabricated Using Layer-by-Layer Assembly of GO and PEDOT:PSS. Sensors and Actuators Physical, 303, 111856.
[5] Wang Y F , Tachibana S , Sekine T . A Printed Flexible Humidity Sensor with High Sensitivity and Fast Response Using a Cellulose Nanofiber/Carbon Black Composite[J]. ACS applied materials & interfaces, 2022(4):14.
[6] Zheng, Y. et al. A flexible humidity sensor based on natural biocompatible silk fibroin films. Adv. Mater. Technol. 6, 2001053 (2021).
